# Glucose transporter GLUT1 influences *Plasmodium berghei* infection in *Anopheles stephensi*

**DOI:** 10.1186/s13071-020-04155-6

**Published:** 2020-06-05

**Authors:** Mengfei Wang, Jingwen Wang

**Affiliations:** Ministry of Education Key Laboratory of Contemporary Anthropology, School of Life Sciences, Fudan University, Shanghai, People’s Republic of China

**Keywords:** *Anopheles stephensi*, *Plasmodium berghei*, Asteglut1, Transcriptome analysis

## Abstract

**Background:**

Sugar-feeding provides energy for mosquitoes. Facilitated glucose transporters (GLUTs) are responsible for the uptake of glucose in animals. However, knowledge of GLUTs function in *Anopheles* spp. is limited.

**Methods:**

Phylogenetic analysis of GLUTs in *Anopheles stephensi* was performed by the maximum likelihood and Bayesian inference methods. The spatial and temporal expression patterns of four *Asteglut* genes were analyzed by qPCR. The function of Asteglut1 was examined using a dsRNA-mediated RNA interference method. Transcriptome analysis was used to investigate the global influence of Asteglut1 on mosquito physiology.

**Results:**

We identified 4 *glut* genes, *Asteglut1*, *Asteglutx*, *Asteglut3* and *Asteglut4* in *An. stephensi*. *Asteglut1*, *Asteglut3* and *Asteglut4* were mainly expressed in the midgut. *Plasmodium berghei* infection differentially regulated the expression of *Asteglut* genes with significant downregulation of *Asteglut1* and *Asteglut4*, while upregulation of *Asteglutx*. Only knocking-down *Asteglut1* facilitated *Plasmodium berghei* infection in *An. stephensi*. This might be due to the accumulation of glucose prior to blood-feeding in dsAsteglut1-treated mosquitoes. Our transcriptome analysis revealed that knockdown of *Asteglut1* differentially regulated expression of genes associated with multiple functional clusters, especially those related to detoxification and immunity. The dysregulation of multiple pathways might contribute to the increased *P. berghei* infection.

**Conclusions:**

Our study shows that Asteglut1 participates in defense against *P. berghei* in *An. stephensi*. The regulation of Asteglut1 on vector competence might through modulating multiple biological processes, such as detoxification and immunity.
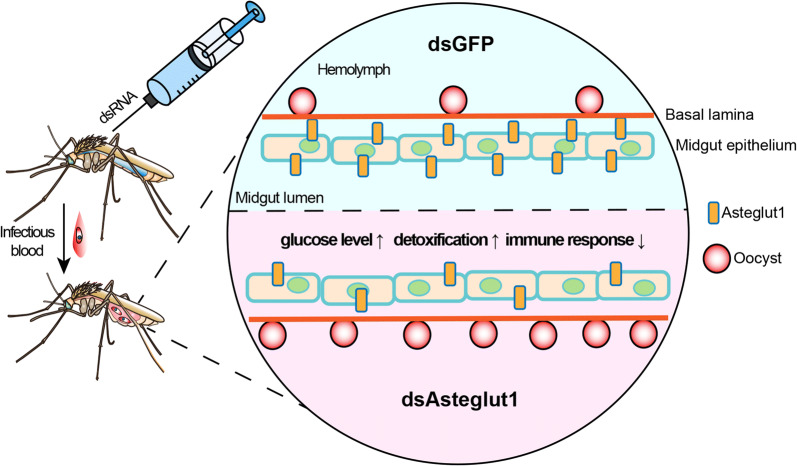

## Background

*Anopheles* mosquitoes are the primary vectors of human malaria that kill over 450,000 people annually [1]. To transmit between mammalian hosts, malaria parasites have to complete multiple development processes in the mosquito including gametogenesis, fertilization, zygote-to-ookinete conversion and oocyst formation [2]. During this process, complicated interactions between *Anopheles* mosquitoes and *Plasmodium* parasites occur [3]. The nutrient availability is one of the key factors that determine the infection outcome [4, 5]. Sugar is a key energy resource that influences survival and fecundity of mosquitoes [6]. It also affects the vector competence [6–10]. Trehalose, the main circulating sugar, is a non-reducing disaccharide composed of two glucose molecules linked by an α-α-1,1-glycosidic bond. It enters cell metabolism after catabolized into glucose [11, 12]. Trehalose transporter AgTreT1 is responsible for the transportation of trehalose from fat body to hemolymph [13]. Knocking-down Tret1 leads to the reduction of hemolymph trehalose and inhibition of *Plasmodium falciparum* infection [13].

Glucose is the primary source of energy for both mosquitoes and *Plasmodium* [14, 15]. During the blood stage and liver stage of malaria infection, *Plasmodium* parasites increase the absorption of glucose in host cells by enhancing the translocation of GLUT1 to the cell membrane [16, 17]. Then these parasites scavenger host glucose by their facilitative hexose transporter (PfHT) [18]. However, the interactions of glucose metabolism between *Anopheles* mosquitoes and *Plasmodium* are still unclear. Only one glucose transporter, *AGAP007752*, is reported to be involved in facilitating *Plasmodium* sporozoites infection in *Anopheles gambiae*, and its knockdown decreased the number of sporozoites in mosquito salivary glands [19–21].

In this study, we identified four *Asteglut* genes in *An. stephensi*. RNAi-mediated silencing of *Asteglut1* specifically increased *P. berghei* infection and significantly elevated the glucose level in mosquito midgut prior to blood-feeding. The accumulation of midgut glucose might modulate multiple biological processes, including detoxification and immunity, which in turn increased parasite infection.

## Methods

### Mosquito rearing and maintenance

*Anopheles stephensi* (strain Hor) was reared at 28 °C and a relative humidity of 80%. Adults were maintained on 2% sucrose solution. Adult female mosquitoes were fed on BALB/c mice for a blood meal.

### *Plasmodium berghei* infection

*Plasmodium berghei* (ANKA strain) parasites expressing GFP constitutively were maintained by passing through BALB/c mice by mosquito biting [22, 23]. When parasitemia of *P. berghei* infected mice rose to 3–6%, mosquitoes which had been starved overnight were allowed to feed on the infected mice for 15 min. Engorged mosquitoes were maintained at 20 °C and un-engorged mosquitoes were removed 24 h post-blood meal. Midguts were dissected and oocyst numbers were counted under a fluorescence microscope at 8 days post-infection. For the melanizaiton assay, midguts were dissected at 8 days post-infection and fixed in 4% formaldehyde for 30 min. Fluorescent oocysts and melanized ookinetes were visualized under a Nikon fluorescence microscope. Pictures were taken using a Nikon confocal microscope (Nikon, Tokyo, Japan).

### Phylogenetic analysis

Sequences were aligned using the default settings in MEGA X software [24]. A phylogenetic tree was constructed using the Maximum Likelihood method based on a bootstrapping method with 1000 replicates. Twenty-five protein sequences were included in the phylogenetic analysis. The sequences obtained from Vectorbase (http://www.vectorbase.org), Flybase (flybase.org) and NCBI database (http://www.ncbi.nlm.nih.gov) were: 4 glucose transporters of *An. stenphensi*, (ASTE005839, ASTE003001, ASTE006385 and ASTE008063), 4 sugar transporters of *An. gambiae* (AGAP007340, AGAP005238, AGAP007752 and AGAP003020), 4 sugar transporters of *Aedes aegypti* (AAEL020018, AAEL006264, AAEL007136 and AAEL010868), 4 sugar transporters of *Drosophila melanogaster* GLUT1 (FBpp0305693), SUT-1 (FBpp0087855), MFST (FBpp0077268) and MFST (FBpp0075675), and 13 glucose transporters of *Homo sapiens*, GLUT1 (NP_006507.2), GLUT2 (XP_011511389.1), GLUT3 (NP_008862.1), GLUT4 (AAI13593.1), GLUT5 (NP_001315548.1), GLUT6 (XP_016869725.1), GLUT7 (XP_011539126.1), GLUT8 (XP_011516904.1), GLUT9 (XP_011512158.1), GLUT10 (XP_011527362.1), GLUT11 (NP_110434.3), GLUT12 (XP_006715412.1) and GLUT14 (XP_024304616.1).

### RNA interference

The *Asteglut* genes were amplified by the corresponding primers: *Asteglut1* (F: 5’-ACA GTA CAA CAG GTG AAG GAA GAG-3’ and R: 5’-GTA ATC CTA CGG TCA CAG CCA AT-3’); *Asteglutx* (F: 5’-GCT GTC AGG AAT CAA TGC CGT CTT-3’ and R:5’-CGC CAC CTC CGT TAC CTC TTG-3’); *Asteglut3* (F: 5’- GCA TTG TTG AGC CAG CCC AAA-3’ and R:5’-CTG CCT CGC CTA GTC CAT TCC-3’); and *Asteglut4* (F:5’- CCA GAT TGC CGA ACC GAT GAC-3’ and R:5’-TCA CCG TGC TCA CCG ATG AT-3’). Primers with the T7 promoter sequence (5’-TAA TAC GAC TCA CTA TAG GG-3’) were used to generate templates for double-stranded RNA (dsRNA). The dsRNAs were synthesized using the MEGAscript RNA kit (Ambio, Invitrogen, Shanghai, China). The plasmid eGFP (BD Biosciences, Shanghai, China) was used as a control. Four-day-old mosquitoes were injected with 69 nl dsRNA (4 μg/μl) using a nanoject II microinjector (Drummond, Philadelphia, USA). The dsRNA-treated mosquitoes were collected two days post-treatment and knockdown efficiency was verified by qPCR as previously described [25].

### RNA isolation, cDNA synthesis and quantitative PCR (qPCR)

Total RNA was extracted from mosquitoes using TRIzol reagent (Sigma-Aldrich, Shanghai, China) according to the manufacturer’s protocol. One µg of total RNA was used to synthesize cDNA using 5× All-in-One MasterMix (AccuRT Genomic DNA Removal Kit; ABM, Shanghai, China). The qPCR was performed using a Roche LightCycler 96 Real Time PCR Detection System using SYBR Green qPCR Master Mix (Biomake, Shanghai, China) according to a previously described protocol [25]. The data were processed and analyzed using the Roche LightCycler 96 software. Ribosomal gene *s7* was used as the internal reference gene.

### Sugar measurement

The glucose and trehalose levels in the mosquito hemolymph and midgut were examined as described [13, 26]. Briefly, 30 μl hemolymph was collected from 10 mosquitoes. Ten midguts were pooled together and homogenized in 30 µl PBS buffer. Thirty microliters of midgut homogenates and hemolymph, respectively, were used for glucose and trehalose measurement; 10 µl was used to measure the glucose level using a Glucose Kit (K-GLUC; Megazyme Bray, Ireland); another 10 µl was treated with trehalase enzyme (K-TREH; Megazyme, Bray, Ireland), and then examined for glucose concentration. Trehalose concentration was calculated as described [13]. The remaining 10 µl was used for genomic DNA extraction and quantification [27]. The concentration of glucose and trehalose were normalized to the amount of genomic DNA, respectively.

### RNA sequencing

Mosquitoes treated with dsAsteglut1 and dsGFP 24 h post-infectious blood meal were collected for RNA sequencing. Four mosquitoes were pooled for one sample and three biological replicates were used from each treatment. Total RNA was extracted using TRIzol® reagent according the manufacturer’s instructions (Sigma-Aldrich) and sent to Majorbio (Shanghai, China) for library construction and sequencing using Illumina HiSeq ×10. Clean data were aligned to the reference genome AsteS1.6 (https://www.vectorbase.org/organisms/anopheles-stephensi). To identify DEGs (differential expression genes) between two groups, the expression level of each transcript was calculated according to the fragments per kilobase of exon per million mapped reads (FPKM) [28]. R statistical package *edgeR* (Empirical analysis of Digital Gene Expression in R; http://www.bioconductor.org/packages/2.12/bioc/html/edgeR.html) was used for differential expression analysis [29]. GO functional enrichment were carried out by Goatools (https://github.com/tanghaibao/Goatools). Both the heat map and Venn map were generated using TBtools software (v0.66831; https://github.com/CJ-Chen/TBtools-Manual) [30]. The pie chart, scatter chart and histogram were created using GraphPad Prism version 6 (GraphPad Software, La Jolla, CA, USA). To demonstrate the similarity across individual biological replicates, principal components analysis (PCA) was performed using the Spotfire DecisionSite for Functional Genomics (DSFG) package (http://spotfire.tibco.com/).

### Statistical analysis

All statistical analyses were performed by GraphPad Prism version 8. Gene expression and sugar levels were compared using the Student’s t-test. Oocyst data were not normally distributed as determined by the Shapiro-Wilk test. Thus, the Mann-Whitney test was used to determine the significance of oocyst intensity in dsRNA-treated mosquitoes.

## Results

### The phylogenetic analysis of glucose transporters in *An. stephensi*

**Fig. 1:**
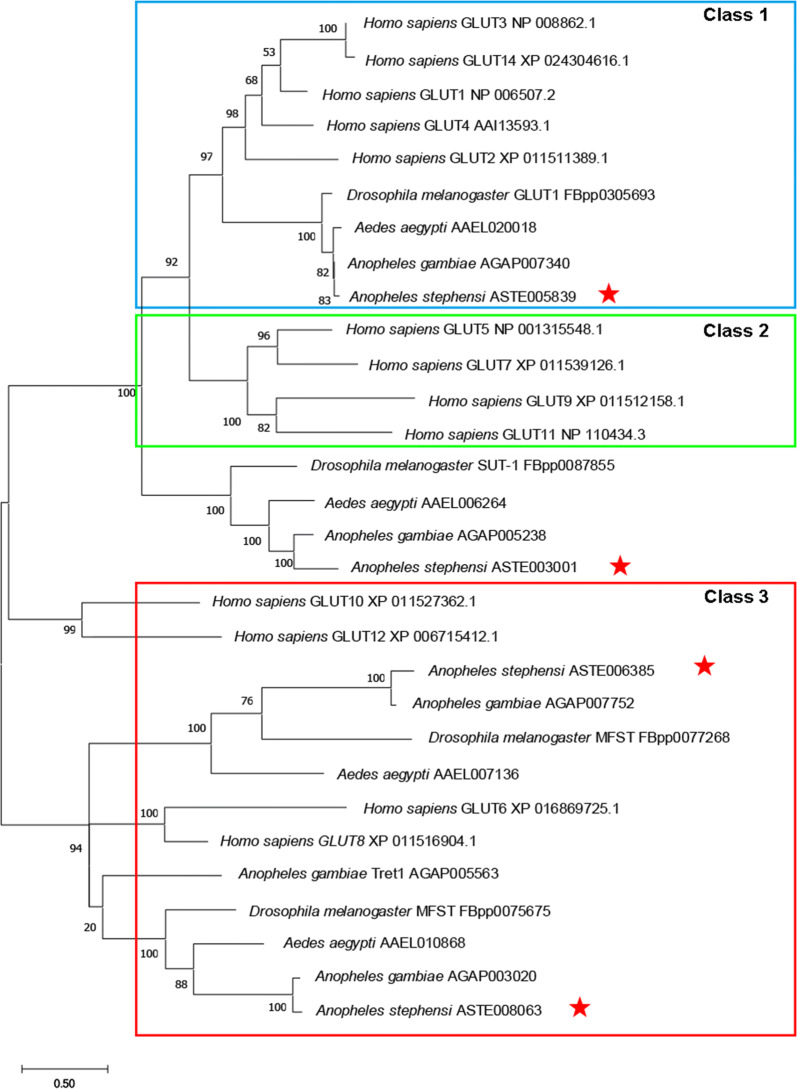
Phylogenetic analysis of glucose transporters (GLUTs) in *An. stephensi*. The genes involved in this study are marked with red stars. The family of GLUT transporters known in humans are divided into three classes; class 1 (blue box), class 2 (green box) and class 3 (red box)

Four genes are annotated as glucose transporters (ASTE005839, ASTE003001, ASTE006385 and ASTE008063) in the Vectorbase of *An. stephensi* (AsteS1.6). To investigate the relationships of these genes between *An. stephensi* and other organisms, a phylogenetic tree was constructed based on the amino acid sequence of *An. gambiae*, *An. stephensi*, *Ae. aegypti*, *D. melanogaster* and *H. sapiens* using the Maximum Likelihood and Bayesian inference phylogenetic analysis (Fig. 1). In *H. sapiens*, Glut transporters can be divided into three classes: class 1 (GLUT1, GLUT2, GLUT3, GLUT4 and GLUT14); class 2 (GLUT5, GLUT7, GLUT9 and GLUT11); and class 3 (GLUT6, GLUT8, GLUT10 and GLUT12) [31–34]. Due to the high similarity between *An. stephensi* ASTE005839, *D. melanogaster* GLUT1 (FBpp0305693) and *H. sapiens* GLUT1 (NP 006507.2), we named ASTE005839 *Asteglut1*. ASTE008063 and ASTE006385 were categorized into GLUT- class 3, and therefore, we named them *Asteglut3* and *Asteglut4*, respectively. ASTE003001 was not phylogenetically related to any GLUT class, therefore, we named this *Asteglutx* (Fig. 1).

### Expression of *Asteglut* genes in *An. stephensi*

**Fig. 2:**
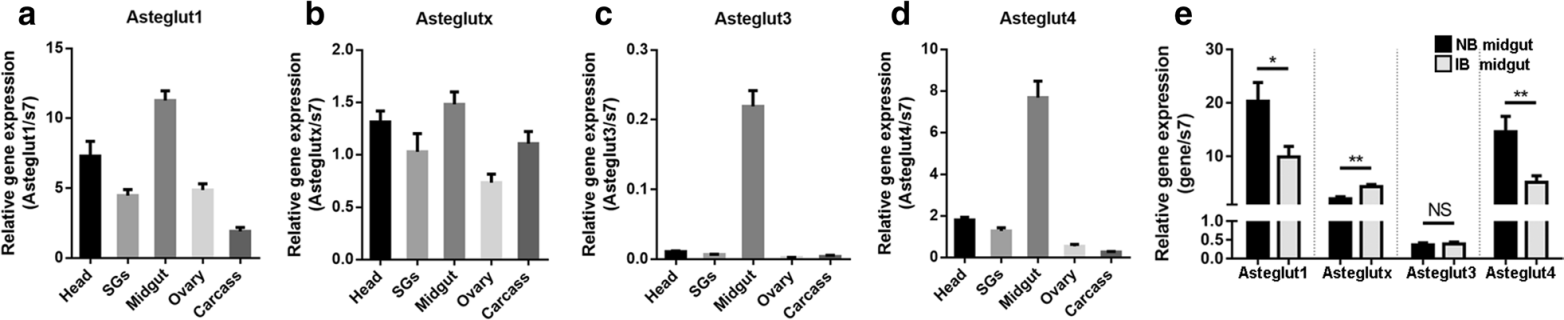
Expression patterns of *Asteglut1*, *Asteglutx*, *Asteglut3* and *Asteglut4* in *An. stephensi*. **a–d** Relative gene expression of *Asteglut1*, *Asteglutx*, *Asteglut3* and *Asteglut4* in the head, salivary glands (SGs), midgut, ovary and carcass 24 h before blood meal, detected by qPCR. **e** Relative gene expression of *Asteglut1*, *Asteglutx*, *Asteglut3* and *Asteglut4* in the midgut 24 h post-normal (NB) and infectious blood (IB) meal. Error bars indicate mean ± SEM (*n* = 5). Results from one of two independent experiments are shown. Significance was determined by Student’s t-test (for details, see Additional file 4: Text S1). *Abbreviations*: NS, non-significant. *P < 0.05, **P < 0.01

To determine the expression pattern of *Asteglut* genes in *An. stephensi*. We analyzed the expression levels of these genes by qPCR in the head, salivary glands, midgut, ovary and carcass 24 h before a blood meal, respectively. *Asteglut1*, *Asteglut3* and *Asteglut4* were mainly localized in the midgut tissue of *An. stephensi* (Fig. 2a, c, d). In addition to the midgut, *Asteglut1* and *Asteglut4* were also expressed in the head and salivary glands (Fig. 2a, d). *Asteglutx* was distributed in all five tissues (Fig. 2b). We next investigated the influence of parasite infection on the expression in the midgut of the four *Asteglut* genes. Asteglut genes were differentially regulated by *P. berghei* 24 h post-infection. (Fig. 2e). *Plasmodium berghei* infection significantly decreased the expression of *Asteglut1* and *Asteglut4* (*t*_(14)_ = 2.585, *P* = 0.0216; *t*_(14)_ = 3.001, *P* = 0.0095), while increased the expression of *Asteglutx* compared to those in normal blood feeding mosquitoes. No influence on *Asteglut3* expression was observed during parasite infection (Fig. 2e, *t*_(14)_ = 0.343, *P* = 0.7369).

### Knockdown of *Asteglut1* facilitates *P. berghei* infection in *An. stephensi*

**Fig. 3:**
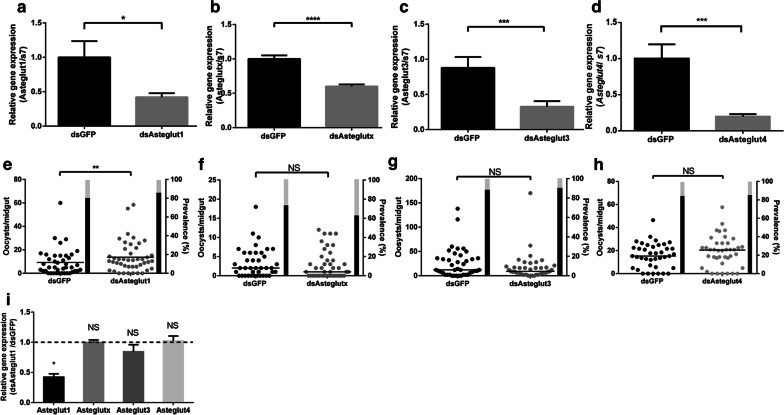
Influence of Asteglut1, Asteglutx, Asteglut3 and Asteglut4 on vector competence. **a–d** Knocking down efficiency of *Asteglut1*, *Asteglutx*, *Asteglut3* and *Asteglut4*. Relative expression levels of *Asteglut1*, *Asteglutx*, *Asteglut3* and *Asteglut4* were normalized to that in dsGFP controls. Ribosomal gene *s7* used as an internal control. Error bars indicate standard error of the mean (*n* = 10). Results from one of three independent experiments are shown. Significance was determined by Student’s t-test. **e**-**h** Oocyst number in dsRNA-treated mosquitoes. Each dot represents an individual mosquito and horizontal lines represent the medians. Results from one of three independent experiments are shown. Significance was determined by Mann-Whitney test. **i** Specificity of dsAsteglut1 treatment. Relative expression level of *Asteglut1*, *Asteglutx*, *Asteglut3* and *Asteglut4* in dsAsteglut1 treated mosquitoes were normalized to that in dsGFPs. Error bars indicate mean ± SEM (*n* = 10). Results from one of three independent experiments are shown. Significance (**a–c**, **d**, **i**) was determined by Student’s t-test (for details, see Additional file 4: Text S1). Significance (**e–h**) was determined by Mann-Whitney test (for details, see Additional file 4: Text S1). *Abbreviations*: NS, non-significant. *P < 0.05, **P < 0.01, ***P < 0.001, ****P < 0.0001

To investigate the role of *Asteglut1*, *Asteglutx*, *Asteglut3* and *Asteglut4* in the capability of *An. stephensi* to transmit *P. berghei*, double-stranded RNA (dsRNA)-mediated silencing strategy was employed. The expression levels of *Asteglut1*, *Asteglutx*, *Asteglut3* and *Asteglut4* were examined two days post-dsRNA treatment. The expression levels of these genes were significantly decreased by 57.8%, 40%, 65% and 80% compared to the dsGFP control, respectively (Fig. 3a–d, *t*_(14)_ = 2.529, *P* = 0.02; *t*_(14)_ = 7.024, *P* < 0.0001; *t*_(14)_ = 3.184, *P* = 0.0002; *t*_(14)_= 3.997, *P* = 0.0013). However, only knockdown of *Asteglut1* significantly increased oocyst number of *P. berghei* (Fig. 3e, *U* = 597, *P* = 0.0067). The dsAsteglutx, dsAsteglut3 and dsAsteglut4 treatments had no apparent effect on the intensity of *P. berghei* infection (Fig. 3f–h, *U* = 746, *P* = 0.3778; *U* = 762, *P* = 0.4748; *U* = 685, *P* = 0.3542). No significant difference of infection prevalence was observed between dsGFP and any dsAsteglut treated mosquitoes (Fig. 3e–h). We next analyzed the knockdown specificity of *Asteglut1* and found this gene was specifically knocked-down (Fig. 3i). Thus, the increasing susceptibility of *An. stephensi* to *P. berghei* infection was due to the knockdown of *Asteglut1*, instead of the compensatory effect of other *Astegluts* (Fig. 3i).

### Knockdown of *Asteglut1* significantly elevates the glucose level in the mosquito midgut

**Fig. 4:**
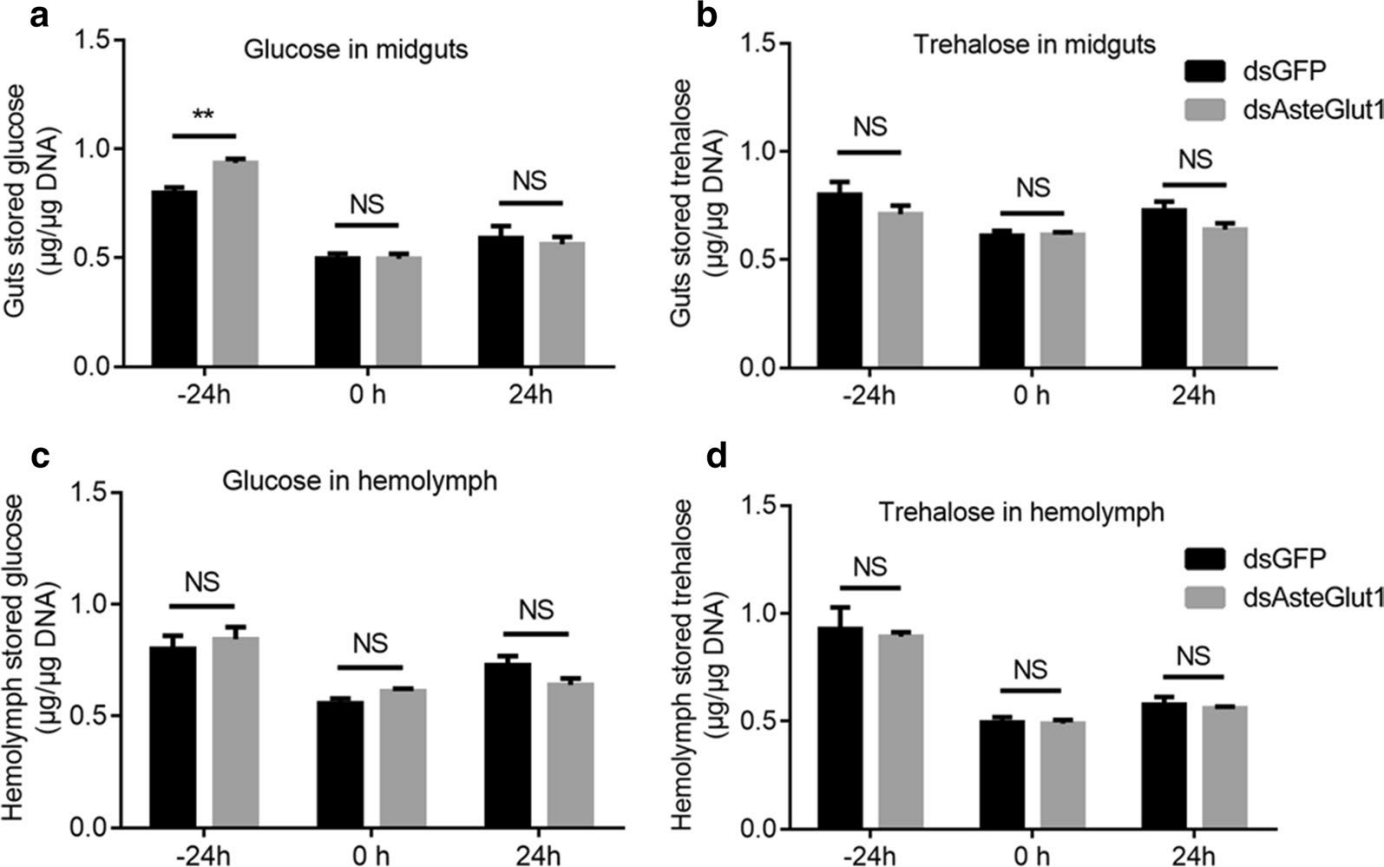
Influence of Asteglut1 on glucose transportation in the mosquito midgut. The relative concentration of glucose (**a**, **c**) and trehalose (**b**, **d**) in the midgut and hemolymph of GFP and Asteglut1 dsRNA- injected *An. stephensi* 24 h prior to (− 24 h), just before (0 h) and 24 h post-blood-feeding (24 h), respectively. Sugar concentration was normalized to genomic DNA extracted from midgut or hemolymph cells. Error bars indicate mean ± SEM (*n* = 5). Significance was determined by Student’s t-test (for details, see Additional file 4: Text S1). *Abbreviation*: NS, non-significant. **P < 0.01

We next analyzed the influence of *Asteglut1* on sugar transportation in *An. stephensi*. The glucose and trehalose levels in the midgut and hemolymph of dsRNA-treated mosquitoes were examined. The glucose level of the *Asteglut1*-knockdown group was significantly higher than that in dsGFP controls 24 h prior to blood-feeding (Fig. 4a, *t*_(8)_ = 4.374, *P* = 0.0024). However, its level in hemolymph is comparable to that in the dsGFP control (Fig. 4c). There was no significant difference between sugar levels in the midgut or hemolymph either just before (0 h) or 24 h post-blood-feeding (Fig. 4, for statistics details, see Additional file 4: Text S1). In addition, knockdown of *Asteglut1* did not change the level of trehalose in the midgut or in hemolymph (Fig. 4b, *t*_(8)_ = 1.299, *P* = 0.2302; *t*_(8)_ = 0.146, *P* = 0.8875; *t*_(8)_ = 1.752, *P* = 0.1180; Fig. 4d, *t*_(8)_ = 0.3585, *P* = 0.7292; *t*_(8)_ = 0.1686, *P* = 0.8703; *t*_(8)_ = 0.4252, *P* = 0.6820). Thus, Asteglut1 might play a role in transportation of glucose, but not trehalose in the mosquito midgut.

### Transcriptional analysis of *Asteglut1*-knockdown mosquitoes

**Fig. 5:**
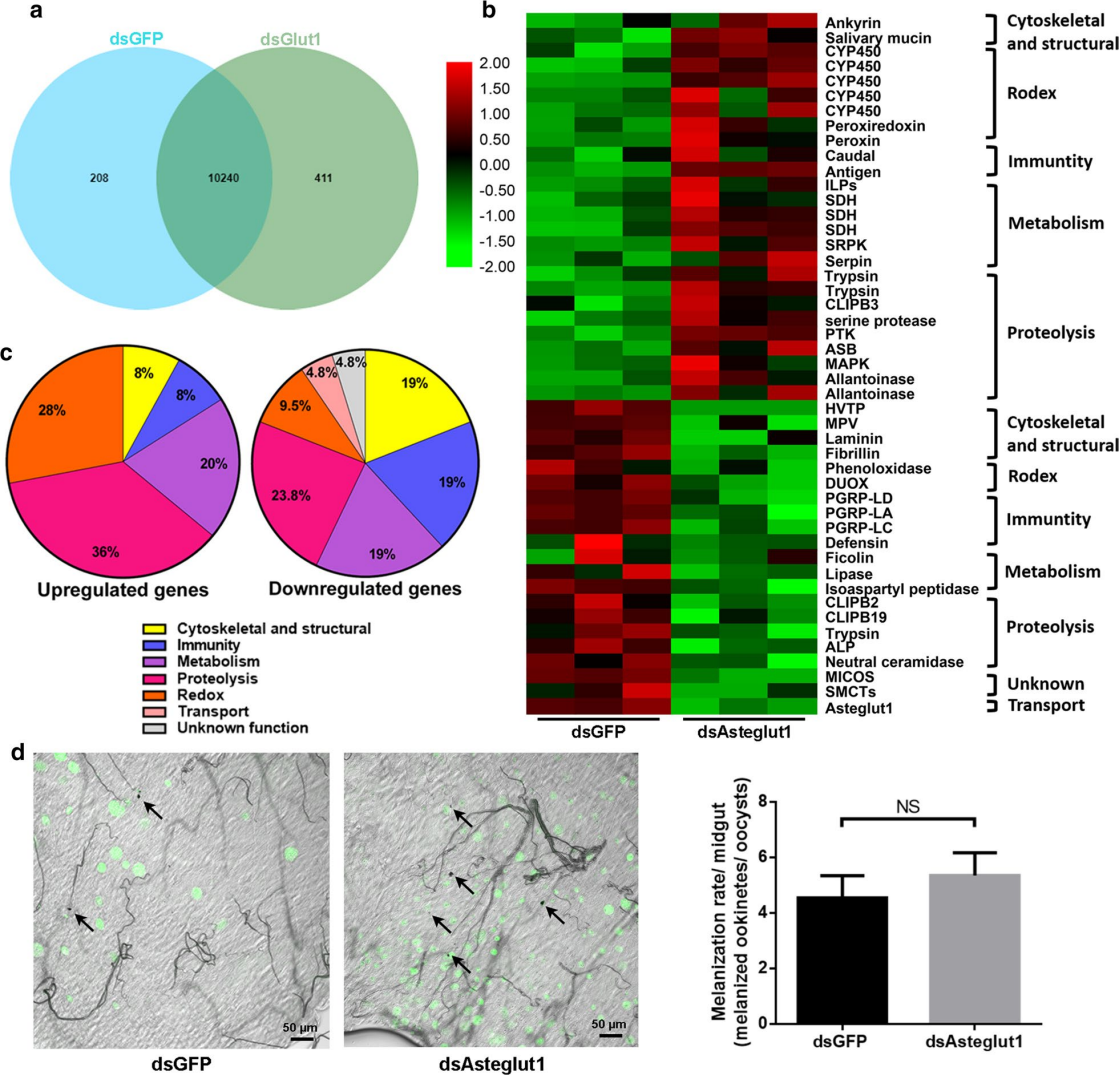
Transcriptome analysis of dsRNA treated *An. stephensi* infected with *P. berghei*. **a** Venn diagram showing overlapping genes between dsGFP- and dsAsteglut1-treated groups. **b** Heatmap of the differentially expressed genes in *An. stephensi* midguts infected with *P. berghei*. Upregulated genes are shown in red; downregulated genes are shown in green (*P* < 0.05; fold change > 2). **c** Pie charts showing the distribution of upregulated genes (left) and downregulated genes (right). **d** Melanization (left panel) and melanization rate (right panel) of *P. berghei* in dsRNA-treated mosquitoes. Live *Plasmodium* oocysts are shown in green and melanized ookinetes are indicated by arrows. Melanization rate was calculated by the ratio of the number of melanized ookinetes to the number of live *Plasmodium* oocysts observed per midgut. Results from one of two independent experiments are shown. Error bars indicate mean ± SEM (*n* = 35). Significance was determined by Student’s t-test (for details, see Additional file 4: Text S1). *Abbreviation*: NS, non-significant. *Scale-bars*: **d**, 50 μm

To explore how Asteglut1 regulated *P. berghei* infection, we performed a transcriptome analysis of the mosquito’s midgut treated with dsAsteglut1 and dsGFP 24 h post-blood meal, respectively. A total of 6 G PE clean sequences were generated by the Illumina HiSeq ×10 (Additional file 1: Table S1). Principal components analysis (PCA) showed a clear separation between dsAsteglut1 and dsGFP treatments (Additional file 2: Figure S1). The Venn diagram shows that the expression of 10,240 genes was overlapped in the two groups (Fig. 5a). A total of 46 genes were differentially expressed (Fig. 5b, Additional file 3: Table S2) with 26 upregulated and 20 downregulated genes. These differentially expressed genes belong to multiple functional clusters, including cytoskeletal and structural, immunity, metabolism, proteolysis, redox, transport and those of unknown function (Fig. 5c).

Among the ‘redox’ functional cluster, five genes encoding cytochrome P450 (CYP450) were upregulated, indicating that detoxification was activated in mosquitoes [35]. The gene encoding peroxiredoxin that controls cytokine-induced peroxide levels in mammalian cells was also significantly upregulated, but the role of this gene in parasite control in mosquitoes is still unknown [36]. We also observed that DUOX (dual oxidase), which is involved in *Plasmodium* elimination, was significantly downregulated in dsAsteglut1-treated mosquitoes (*P* < 0.0001) [37]. It is highly possible that the reduction of DUOX expression might render mosquitoes more permissive to *P. berghei* infection.

The CLIP (class of serine proteases) family are involved in the melanization of *P. berghei* in *An. gambiae* [38]. Two CLIP genes, *clip2* and *clip19*, were significantly downregulated in dsAsteglut1 treated mosquitoes, while *clipb3* was upregulated compared to dsGFP mosquitoes (*P* = 0.0087, *P* = 0.0034) [38, 39]. Next, we examined whether the increasing parasite infection could be due to the dysregulation in mosquito melanization. Midguts of mosquitoes treated with dsRNA 8 days post-infection were collected and melanization was visualized microscopically. We found that the number of melanized ookinetes increased with the number of oocysts (Fig. 5d, *t*_(68)_ = 0.707, *P* = 0.482). Thus, there was no significant difference in the melanization rate between dsAsteglut1-treated and the dsGFP control group.

Five immune related genes were differentially regulated. *Caudal*, the negative regulator of Imd pathway was significantly upregulated (*P* = 0.0337) [40], while the peptidoglycan recognition proteins, *pgrp-la*, -*lc*, -*ld*, and the antimicrobial peptides, *defensin* were significantly downregulated (*P* = 0.0111, *P* = 0.0378, *P* = 0.0022) [25, 40–43]. These results indicate that *Asteglut1* might control parasite infection by regulating mosquito immune responses.

## Discussion

The glucose transporter family, functionally conserved from insects to mammals, is responsible for the transportation of glucose across the cell membrane [44]. In mammals, GLUT1 is one of the earliest cloned membrane transporters and has been extensively investigated in the past half century [45]. It is ubiquitously expressed in the skeletal, muscle, heart, and other tissues, but predominantly functions in erythrocytes and the blood-brain barrier [32, 45, 46]. In *An. stephensi*, *Plasmodium* undergoes a drastic reduction during the early stage of their infection in mosquitoes. In the entire *Plasmodium* life-cycle (in both human and mosquito hosts), parasite number is the lowest at the oocyst stage and then quickly increases with thousands of sporozoites produced per oocyst [47]. For this reason, we focused on the interactions between the midgut stages of parasites and mosquitoes, aiming to find a possible target for vector control. We identified 4 *glut* genes, *Asteglut1*, *Asteglut3*, *Asteglutx* and *Asteglut4*. They have distinct expression patterns, suggesting their potential different roles in glucose transportation. Knockdown of *Asteglut1* increased the glucose level in the midgut, suggesting its role in maintaining the homeostasis of intestinal glucose. However, we did not observe significant changes of glucose and trehalose levels in the hemolymph. It is highly possible that the sugar level in hemolymph is controlled by multiple factors and functional redundancy exists between members of the *Asteglut* family.

In addition, we also found that knockdown of *Asteglut1* influences the mosquito’s susceptibility to *P. berghei* infection. In agreement with our findings, GLUT1 is involved in the regulation of pathogen infection in mammals. GLUT1 is a natural receptor of T-lymphotropic virus (HTLV) that facilitates the invasion of HTLV in human cells [48]. GLUT1 is also involved in the regulation of CD4^+^ T cell function in humans. Knocking out GLUT1 in CD4^+^ T cells reduces glucose uptake and glycolysis, and also impairs the growth, proliferation, survival and differentiation of these cells [49]. In plants, the expression of sugar transporter (SWEET) is induced by bacterial and fungal infection. Knockout of SWEET limits the growth of these pathogens [50]. In *An. stephensi*, invasion of *P. berghei* into salivary glands induces the expression of the glucose transporter *AGAP007752*. Its knockdown decreased the number of sporozoites in mosquito salivary glands [19–21].

Asteglut1 help to defense against *P. berghei* might occur through regulating midgut glucose level. The accumulation of glucose in the midgut when *Asteglut1* is knocked-down might change multiple biological processes, which effect synergistically to increase parasite infection. Our transcriptome analysis reveals that a considerable number of upregulated genes are cytochrome P450s, which are responsible for detoxification [51]. The upregulation of cytochrome P450 genes in dsAsteglut1-treated mosquitoes indicate that these mosquitoes might suffer more toxicity than that in control [51, 52]. In addition to catabolizing xenobiotics, the cytochrome P450s are also involved in the anabolism and catabolism of hormones [53]. For example, cytochrome P450s are involved in the biosynthesis of 20-hydroxyecdysone (20E) from cholesterol [54]. The steroid hormone 20E not only promotes oogenesis in mosquitoes, but also facilitates *Plasmodium* infection [55]. Thus, the elevated levels of P450 gene expression might be responsible for increased parasite infection.

The CLIP family members function as either activators or suppressors of melanization that are responsible for elimination of *P. berghei* in *An. gambiae* [38]. Although three *clips* genes are differentially regulated in dsAsteglut1-treated mosquitoes, we did not observe any difference in melanization rates between dsGFP and dsAsteglut1 treated mosquitoes. This result suggests that these CLIPs might function differently from classical CLIPS. Further investigation of their function is required.

We also noticed the significant induction of *caudal*, and reduction of *pgrps*, *pgrp-la*, *pgrp-lc*, *pgrp-ld*, and the antimicrobial peptides, *defensin* in dsAsteglut1-treated mosquitoes, suggesting that *Asteglut1* might be involved in regulation of immune responses [25, 40, 56]. PGRP-LA is a receptor of the mosquito immune deficiency pathway (Imd) [41]. It helps to control the homeostasis of gut microbiota and parasite infection in *An. stephensi* [57]. PGRP-LC is the primary receptor of the Imd pathway. Silencing PGRP-LC blocks the synthesis of downstream immune effectors, which in turn increases parasite infection [42]. Different from PGRP-LA and LC, PGRP-LD is a negative regulator of the immune signaling pathway. However, knockdown of PGRP-LD similarly increased susceptibility of *An. stephensi* to *P. berghei* infection through impairing the integrity of the peritrophic matrix. The compromised peritrophic matrix structure results from the reduction of gut microbiota in the absence of protection by PGRP-LD [25]. Thus, the downregulation of *pgrp-la*, *pgrp*-*lc* and *pgrp*-*ld* all lead to increasing susceptibility of the *Anopheles* mosquito to *Plasmodium* infection [25, 42, 57]. However, how *Asteglut1* regulates the Imd pathway needs to be investigated in the future.

## Conclusions

**Fig. 6:**
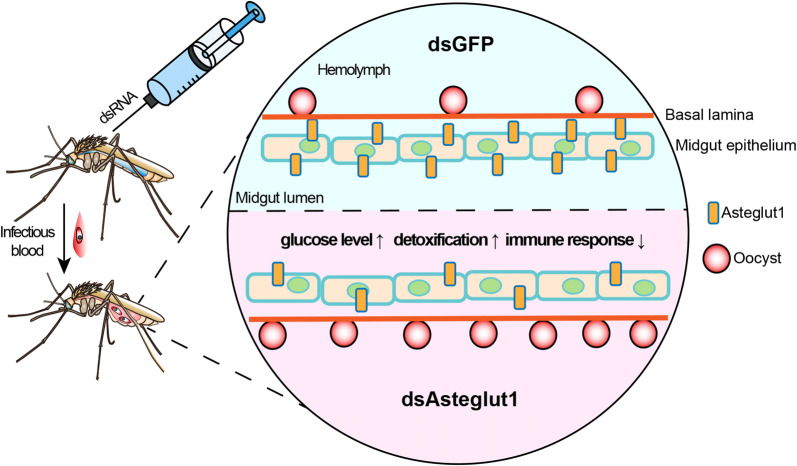
Model of Asteglut1 function in *An. stephensi*. Abrogation of *Asteglut1* significantly increases the susceptibility of *An. stephensi* to *P. berghei* infection. The regulation of Asteglut1 on vector competence might through modulating multiple biological processes, especially detoxification and immunity

In summary, we identified 4 GLUT members in *An. stephensi* and found Asteglut1 participates in the defense against *P. berghei* infection. The regulation of vector competence by *Asteglut1* might occur through modulating multiple biological processes, especially detoxification and immunity (Fig. 6). Our findings pave the way for further understanding as to how sugar transporters regulate vector-parasite interactions and will help to explore potential new targets for vector control.

## Supplementary information

**Additional file 1: Table S1.** Summary of RNA-sequencing data generated using Illumina Hiseq platform.

**Additional file 2: Figure S1.** Principal components analysis (PCA) of transcriptome profiles produced by RNA-seq. First principal component (PC1) is shown on x-axis while the second principal component (PC2) is shown on y-axis. Percentages denote the amount of variance explained by each different PC.

**Additional file 3: Table S2.** List of significantly differentially expressed genes.

**Additional file 4: Text S1.** Details of statistical analyses in this study.

## Data Availability

All data generated or analyzed during this study are included in this published article and its additional files. Raw RNA-seq sequencing data has been uploaded to the National Center for Biotechnology Information’s Sequence Read Archive (Accession no. PRJNA597441).

## Abbreviations

Asteglut: glucose transporter in *Anopheles stephensi*
CYP450: cytochrome P450
DUOX: dual oxidase
CLIP: class of serine proteases
