# Supplementary material for: Glucose transporter GLUT1 influences Plasmodium berghei infection in Anopheles stephensi
Source: Parasit Vectors. 2020 Jun 5;13:285. doi: 10.1186/s13071-020-04155-6 (PMC7275331; doi:10.1186/s13071-020-04155-6)
Supplement: Supplementary file 1 — Additional file 1: Table S1. Summary of RNA-sequencing data generated using Illumina Hiseq platform. [file 13071_2020_4155_MOESM1_ESM.docx]

**Additional file 1: Table S1.** Summary of RNA-sequencing data generated using Illumina Hiseq platform.

| # | Biological treatment | Raw reads | Clean reads |
| --- | --- | --- | --- |
| 1 | dsGFP | 56091442 | 55685716 |
| 2 | dsGFP | 60682804 | 60154312 |
| 3 | dsGFP | 55811924 | 55422812 |
| 1 | dsAsteglut1 | 56755486 | 56322158 |
| 2 | dsAsteglut1 | 60465040 | 59968780 |
| 3 | dsAsteglut1 | 52767086 | 52344266 |
