# Supplementary figures and images for: Glucose transporter GLUT1 influences Plasmodium berghei infection in Anopheles stephensi
Source: Parasit Vectors. 2020 Jun 5;13:285. doi: 10.1186/s13071-020-04155-6 (PMC7275331; doi:10.1186/s13071-020-04155-6)

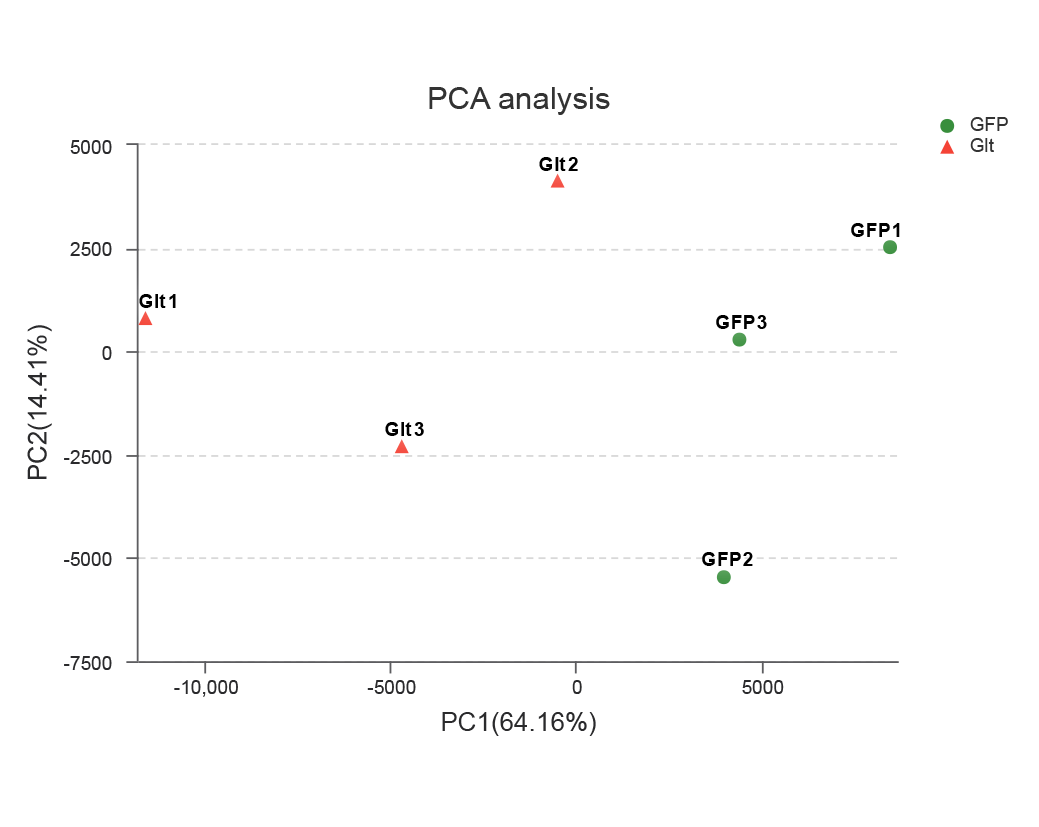

Supplement: Supplementary file 2 — Additional file 2: Figure S1. Principal components analysis (PCA) of transcriptome profiles produced by RNA-seq. First principal component (PC1) is shown on x-axis while the second principal component (PC2) is shown on y-axis. Percentages denote the amount of variance explained by each different PC. [file 13071_2020_4155_MOESM2_ESM.tif]
