# Supplementary material for: Glucose transporter GLUT1 influences Plasmodium berghei infection in Anopheles stephensi
Source: Parasit Vectors. 2020 Jun 5;13:285. doi: 10.1186/s13071-020-04155-6 (PMC7275331; doi:10.1186/s13071-020-04155-6)
Supplement: Supplementary file 4 — Additional file 4: Text S1. Details of statistical analyses in this study. [file 13071_2020_4155_MOESM4_ESM.docx]

**Additional file 4: Text S1.** Details of statistical analyses in this study.

Fig. 2e:

Group 1 vs Group 2: Student’s *t* test: *t*_(14)_ = 2.585, *P* = 0.0216

Group 3 vs Group 4: Student’s *t* test: *t*_(14)_ = 4.096, *P* = 0.0011

Group 5 vs Group 6: Student’s *t* test: *t*_(14)_ = 0.343, *P* = 0.7369

Group 7 vs Group 8: Student’s *t* test: *t*_(14)_ = 3.001, *P* = 0.0095

Fig. 3a:

Group 1 vs Group 2: Student’s *t* test: *t*_(14)_ = 2.529, *P* = 0.02

Fig. 3b:

Group 1 vs Group 2: Student’s *t* test: *t*_(14)_ = 7.024, *P*<0.0001

Fig. 3c:

Group 1 vs Group 2: Student’s *t* test: *t*_(14)_ = 3.184, *P*=0.0002

Fig. 3d:

Group 1 vs Group 2: Student’s *t* test: *t*_(14)_= 3.997, *P* = 0.0013

Fig. 3e:

Group 1 vs Group 2: Mann-Whitney test: *U* = 597, *P* = 0.0067

Fig. 3f:

Group 1 vs Group 2: Mann-Whitney test: *U* = 746, *P* = 0.3778

Fig. 3g:

Group 1 vs Group 2: Mann-Whitney test: *U* = 762, *P* = 0.4748

Fig. 3h:

Group 1 vs Group 2: Mann-Whitney test: *U* = 685, *P* = 0.3542

Fig. 3i:

dsAsteglut1 vs dsGFP

Group 1: Student’s *t* test: *t*_(14)_ = 2.529, *P* = 0.02

Group 2: Student’s *t* test: *t*_(18)_ = 0.0286, *P* = 0.9775

Group 3: Student’s *t* test: *t*_(18)_ = 0.8332, *P* = 0.4156

Group 4: Student’s *t* test: *t*_(14)_ = 0.2053, *P* = 0.8403

Fig. 4a:

Group 1 vs Group 2: Student’s *t* test: *t*_(8)_ = 4.374, *P* = 0.0024

Group 3 vs Group 4: Student’s *t* test: *t*_(8)_ = 0.033, *P* = 0.9745

Group 5 vs Group 6: Student’s *t* test: *t*_(8)_ = 0.455, *P* = 0.6611

Fig. 4b:

Group 1 vs Group 2: Student’s *t* test: *t*_(8)_ = 1.299, *P* = 0.2302

Group 3 vs Group 4: Student’s *t* test: *t*_(8)_ = 0.146, *P* = 0.8875

Group 5 vs Group 6: Student’s *t* test: *t*_(8)_ = 1.752, *P* = 0.1180

Fig. 4c:

Group 1 vs Group 2: Student’s *t* test: *t*_(8)_ = 0.524, *P* = 0.6144

Group 3 vs Group 4: Student’s *t* test: *t*_(8)_ = 2.230, *P* = 0.0563

Group 5 vs Group 6: Student’s *t* test: *t*_(8)_ = 1.764, *P* = 0.1157

Fig. 4d:

Group 1 vs Group 2: Student’s *t* test: *t*_(8)_ = 0.3585, *P* = 0.7292

Group 3 vs Group 4: Student’s *t* test: *t*_(8)_ = 0.1686, *P* = 0.8703

Group 5 vs Group 6: Student’s *t* test: *t*_(8)_ = 0.4252, *P* = 0.6820

Fig. 5d:

Group 1 vs Group 2: Student’s *t* test: *t*_(68)_ = 0.707, *P* = 0.482
